# Gene Sequencing for Pathogenic Variants Among Adults With Breast and Ovarian Cancer in the Caribbean

**DOI:** 10.1001/jamanetworkopen.2021.0307

**Published:** 2021-03-01

**Authors:** Sophia H. L. George, Talia Donenberg, Cheryl Alexis, Vincent DeGennaro, Hedda Dyer, Sook Yin, Jameel Ali, Raleigh Butler, Sheray N. Chin, DuVaughn Curling, Dwight Lowe, John Lunn, Theodore Turnquest, Gilian Wharfe, Danielle Cerbon, Priscila Barreto-Coelho, Matthew P. Schlumbrecht, Mohammad R. Akbari, Steven A. Narod, Judith E. Hurley

**Affiliations:** 1 Division of Gynecologic Oncology, Department of Obstetrics, Gynecology and Reproductive Sciences, University of Miami, Miami, Florida; 2 Sylvester Comprehensive Cancer Center, Miami, Florida; 3 Leonard Miller School of Medicine, University of Miami, Miami, Florida; 4 Department of Genetics, University of Miami, Miami, Florida; 5 Faculty of Medical Sciences, University of West Indies-Cave Hill, Barbados; 6 Innovation Health International, Haiti; 7 Ross University School of Medicine, Commonwealth of Dominica (now in Barbados); 8 Cayman Islands Cancer Society, Grand Cayman, Cayman Islands; 9 St. James Medical Complex, Northwest Regional Health Authority, Port-of-Spain, Trinidad and Tobago; 10 Princess Margaret Hospital, University of the West Indies, School of Clinical Medicine and Research, Nassau, Bahamas; 11 Department of Pathology, University of West Indies-Mona, Kingston, Jamaica; 12 Division of Medical Oncology, Department of Medicine, University of Miami, Miami, Florida; 13 Women’s College Research Institute, Women’s College Hospital, University of Toronto, Toronto, Canada

## Abstract

**Importance:**

Rates of breast and ovarian cancer are high in the Caribbean; however, to date, few published data quantify the prevalence of inherited cancer in the Caribbean population.

**Objective:**

To determine whether deleterious variants in genes that characterize the hereditary breast and ovarian cancer syndrome are associated with the development of breast and ovarian cancer in the English- and Creole-speaking Caribbean populations.

**Design, Setting, and Participants:**

This multisite genetic association study used data from germline genetic test results between June 2010 and June 2018 in the Bahamas, Cayman Islands, Barbados, Dominica, Jamaica, Haiti, and Trinidad and Tobago. Next-generation sequencing on a panel of 30 genes and multiplex ligation-dependent probe amplification (*BRCA1* and *BRCA2*) were performed. Medical records were reviewed at time of study enrollment. Women and men diagnosed with breast and ovarian cancer with at least 1 grandparent born in the participating study sites were included; 1018 individuals were eligible and consented to participate in this study. Data were analyzed from November 4, 2019, to May 6, 2020.

**Exposures:**

Breast and/or ovarian cancer diagnosis

**Main Outcomes and Measures:**

Rate of inherited breast and ovarian cancer syndrome and spectrum and types of variants.

**Results:**

Of 1018 participants, 999 (98.1%) had breast cancer (mean [SD] age, 46.6 [10.8] years) and 21 (2.1%) had ovarian cancer (mean [SD] age, 47.6 [13.5] years). Three individuals declined to have their results reported. A total of 144 of 1015 (14.2%) had a pathogenic or likely pathogenic (P/LP) variant in a hereditary breast and ovarian cancer syndrome gene. A total of 64% of variant carriers had P/LP variant in *BRCA1*, 23% in *BRCA2*, 9% in *PALB2* and 4% in *RAD51C*, *CHEK2, ATM, STK11* and *NBN*. The mean (SD) age of variant carriers was 40.7 (9.2) compared with 47.5 (10.7) years in noncarriers. Individuals in the Bahamas had the highest proportion of hereditary breast and ovarian cancer (23%), followed by Barbados (17.9%), Trinidad (12%), Dominica (8.8%), Haiti (6.7%), Cayman Islands (6.3%), and Jamaica (4.9%). In Caribbean-born women and men with breast cancer, having a first- or second-degree family member with breast cancer was associated with having any *BRCA1* or *BRCA2* germline variant (odds ratio, 1.58; 95% CI, 1.24-2.01; *P* < .001). A *BRCA1* vs *BRCA2* variant was more strongly associated with triple negative breast cancer (odds ratio, 6.33; 95% CI, 2.05-19.54; *P* = .001).

**Conclusions and Relevance:**

In this study, among Caribbean-born individuals with breast and ovarian cancer, 1 in 7 had hereditary breast and ovarian cancer. The proportion of hereditary breast and ovarian cancer varied by island and ranged from 23% in the Bahamas to 4.9% in Jamaica. Each island had a distinctive set of variants.

## Key Points

**Question:**

What proportion of patients in the Caribbean who develop breast or ovarian cancer carry deleterious variants?

**Findings:**

This genetic association study included 1018 adult women and men with breast and ovarian cancer, of which 144 individuals had a pathogenic variant in a moderate- to high-risk gene. This finding was consistent with high rates of premenopausal breast cancer in Black women with Caribbean ancestry.

**Meaning:**

Results of this study suggest that in people with Caribbean ancestry diagnosed with breast or ovarian cancer, 1 in 7 will have an actionable pathogenic variant, which may lead to use of targeted therapies and precision prevention approaches.

## Teaser

This genetic association study assesses the prevalence of pathogenic gene variants among adults with breast and ovarian cancer in 7 Caribbean nations.

## Introduction

The Caribbean is an archipelago of island nations that is home to 40 million people. The population is predominantly of African descent with genetic admixture of Indigenous, Asian, Indian, European and Middle Eastern immigrants. .Breast cancer is the leading case of cancer-related deaths in Caribbean women and in some countries disproportionately affects young women (age 45-59 years).^1,2,3,4^

In the US and Western Europe, 5% to 10% of patients with breast cancers (30% of which are early onset) and 25% of patients with ovarian cancers carry a variant in a cancer predisposing gene.^5,6,7,8^ In West Africa, approximately 14% of patients with breast cancer have a pathogenic or likely pathogenic (P/LP) variant in a known hereditary breast and ovarian cancer gene.^9,10^ Anecdotal information pointed to a high incidence of inherited breast cancer in the Bahamas. This information was confirmed with testing for variants in the *BRCA1* and *BRCA2* genes which revealed that 23% of patients had a P/LP variant in *BRCA1* or *BRCA2*.^11,12,13,14^ Seven recurrent variants were identified, 5 in *BRCA1* and 2 in *BRCA2*.^12^

Given the high rates of breast and ovarian cancer in the Caribbean, and the relatively young age at presentation, we sought to determine the rate of inherited breast and ovarian cancer in select countries in the Caribbean and to identify the spectrum of variants across the region. We characterized the odds of having a P/LP variant when diagnosed at an early age for this population. We therefore conducted multigene testing for hereditary breast and ovarian cancer genes in patients from 7 Caribbean countries.

## Methods

### Cohort

The Caribbean Women’s Cancer Study is a cross-sectional study of patients with invasive breast cancer and/or ovarian cancer born in the Caribbean. The present study was conducted from June 2010 to June 2018, with data analyzed between 2019-2020. This study followed the Strengthening the Reporting of Genetic Association Studies (STREGA) reporting guideline for genetic association studies.^15^ A total of 1018 participants were enrolled. Institutional review board approval was obtained at the Ministries of Health in The Bahamas, Barbados and Cayman Islands, Dominica, and Haiti. Institutional board review approval was also obtained from the University of West Indies, Mona, Jamaica, the North West Regional Health Authority of Trinidad and Tobago (Trinidad), and the University of Miami. The study was led by a local principal investigator (J.A. [Trinidad and Tobago]; J.L. and J.H. [Bahamas]; H.D. [Dominica]; C.A. [Barbados]; G.W. [Jamaica]; S.Y. [Cayman]; and V.D.G.[Haiti]). Participants were identified by treating physicians, local cancer societies, hospital and pathology records, and the outpatient oncology clinical records of the individual islands. Participants were also recruited through media outlets, including radio, newspaper, and television advertisements. In brief, inclusion criteria were a pathologic diagnosis of breast cancer and/or ovarian cancer; individuals needed to have at least 1 grandparent born in 1 of the 7 participating countries: the Bahamas, the Cayman Islands, Barbados, Dominica, Haiti, Jamaica, and Trinidad and Tobago; ability to provide consent, and the ability to provide a saliva specimen. All participants gave informed consent and underwent individual pretesting genetic counseling by a genetic counselor with a master’s degree in genetic counseling (T.D.). The consent form included presentation of results in publication, and participants authorized the study team to obtain and review their cancer-related medical records and to share their study results with their oncologists.

### Data Collection

Data collected included patient demographic characteristics: age at diagnosis, contact information for return of results, current age, self-identified race/ethnicity, sex, 3-generation family pedigree, age of menarche, age of menopause, age of first pregnancy, number of pregnancies, number of siblings, birth order, year of birth, age at cancer diagnosis, current body mass index (BMI [calculated as weight in kilograms divided by height in meters squared]), stage at cancer diagnosis, mode of diagnosis, and tumor characteristics: tumor grade, histology, and ER/PR/*ERBB2* (formerly *HER2*) immunohistochemistry.

### Genetics and Genomics

Following written informed consent and individual genetic counseling, a saliva sample was obtained using a DNA sample collection kit (Oragene OG-250; DNAGenotek). Genomic DNA was isolated following the manufacturer's instructions. All samples underwent next-generation sequencing and multiplex ligation–dependent probe amplification. These analyses enabled all classes of variants including point variants, small insertions, and small and large genomics deletions or duplications. Specifically, the study cohort was initially screened for *BRCA1*, *BRCA2*, *PALB2* and *RAD51* (phase 1) variants across introns, exons, 5′UTR and 3′UTR. Following access to multigene panel testing, study participants in Barbados, Cayman Islands, Dominica, and Haiti underwent full NGS of 30 genes (phase 2): for *BRCA1, BRCA2, MLH1, MSH2, MSH6, PMS2, EPCAM, MUTYH, APC, STK11, PALB2, MITF, BAP1, CDKN2A, TP53, BMPR1A, SMAD4, POLD1, POLE1, CHEK2, PTEN, CDH1, BRIP1, CDK4, GREM1, RAD51C, RAD51D, PMS2, NBN* and *BARD1* (Color Genomics).^16,17^ Study participants in Jamaica and Trinidad with a family history of breast or ovarian cancer, age less than 40 years, and negative test results for *BRCA1, BRCA2, PALB2*, and *RAD51C* in phase 1 were reassessed by the multipanel test. Results were reported to participants and oncologists as pathogenic (the variant directly contributes to the development of disease), likely pathogenic (a high likelihood [>90% certainty] that the variant is disease causing), and/or variant of unknown significance (VUS). Probands were referred to the study genetic counselor for further assessment, discussion of additional risks of secondary cancers, opportunities for risk reduction, and risks to family members.

### Statistical Analysis

All statistical calculations were performed with IBM SPSS statistical software version 25 (IBM Corp) and Stata version 14 IC (StataCorp LLC). Participants were described in terms of age, island of origin, tumor characteristics, ER, PR, *ERBB2*/neu, personal history of cancer and family history (number of relatives with breast, ovarian. and/or endometrial cancer and relationship to the participant). Analysis of categorical variables was based on 2-tailed χ^2^ tests, with Pearson continuity correction. Continuous variables were compared with independent *t* test, 1-way analysis of variance as appropriate, assuming normal distribution, and Wilcoxon rank sum test if not normally distributed. Univariable logistic regression models were used to calculate odds ratios (ORs) and 95% CIs.^18^ All tests were 2-sided, with significance set at *P* = .05.

## Results

### Demographic Characteristics

In total, 1018 participants with breast and ovarian cancer were enrolled in the study, representing 996 women, 21 of whom had ovarian cancer, and 3 men with breast cancer. Demographic characteristics are listed in Table 1. Detection of breast cancer through screening mammogram was uncommon as 86% of patients with breast cancer self-detected a breast mass and then sought medical attention (eTable 1 in the Supplement). A total of 63% of the women diagnosed with breast cancer were premenopausal. The mean (SD) age at diagnosis of breast cancer was 46.6 (10.8) years and for ovarian cancer, 47.6 (13.5) years. We saw in both Trinidad and Tobago (study age of 42.9 years vs registry age of 56 years) and Barbados (study age of 46.5 years vs registry age of 57.9 years) discrepancies of study age and population-based cancer registries.^19,20^ Among patients with documented tumor stage at diagnosis, 33.4% (203 of 607) were diagnosed at stage III and 5.9% had stage IV disease. The highest percentage of advanced stage disease was found in Haiti where 64.7% (44 of 68) had stage III/IV while the Cayman Islands had the fewest (11%). Immunohistochemistry was not routinely performed on specimens in Dominica and was inconsistently done in Haiti. Among patients with breast cancer with available immunohistochemical tumor markers (ER, PR, and *ERBB2*), 59.6% (344 of 577) of breast cancers were ER positive and 24.1% (129 of 536) were triple negative (TNBC). Twenty-one percent of the tumors tested were *ERBB2*/neu positive (108 of 497), ranging from 26.3% in Jamaica to 13.5% in Cayman Islands. The rate of TNBC was 24.1% overall.

**Table 1.** Demographic, Clinical, and Pathologic Features of Cohort at Time of Diagnosis

Characteristic	Country of birth, No./total No. (%)							
	Bahamas	Barbados	Cayman	Dominica	Haiti	Jamaica	Trinidad	Total
Participants, No.	247	92	62	61	75	183	298	1018
Cancer								
Breast	243/247 (98.4)	90/92 (97.8)	57/62 (91.9)	60/61 (98.4)	75/75 (100)	182/183 (99.5)	292 /298 (98)	999/1018 (98.1)
Ovarian	5/247 (2)	2/92 (2.2)	5/62 (8.1)	1/61 (1.6)	0	1/183 (0.5)	7 (2.3)	21/1018 (2.1)
Age at diagnosis, mean (SD)								
BC	44.9 (10.54)	46.5 (10.43)	52.5 (12.23)	52.1 (10.49)	52.1 (11.01)	48.8 (10.74)	42.9 (9.05)	46.6 (10.81)
OC	51.6 (10.33)	43.0 (21.21)	45.2 (13.84)	42	NR	39	50.0 (16.72)	47.6 (13.45)
BMI, mean (SD)	29.8 (6.9)	29.7 (5.8)	29.3 (6)	28.04 (6.9)	28.7 (6.3)	29.08 (6.3)	28.5 (6.56)	29.1 (6.5)
BC stage								
I	3/19 (15.8)	17/54 (31.5)	12/28 (42.9)	7/27 (25.9)	1/68 (1.5)	34/141 (24.1)	58/270 (21.5)	132/607 (21.7)
II	8/19 (42.1)	12/54 (22.2)	13/28 (46.4)	10/27 (27)	12/68(17.6)	54/141 (38.3)	118/270 (43.7)	236/607 (38.9)
III	7/19 (36.8)	10/54 (18.5)	3/28 (10.7)	10/27 (27)	38/68 (55.9)	48/141 (34)	83/270 (30.7)	203/607 (33.4)
IV	1/19 (5.3)	2/54 (3.7)	0	0	6/68 (8.8)	5/141 (3.5)	11/270 (4.1)	36/607 (5.9)
ER positive	10/18 (55.6)	53/75 (70.7)	30/45 (66.7)	20/24 (83.3)	11/21 (52.4)	93/143 (65)	127/251 (50.6)	344/577 (59.6)
*ERBB2* (formerly *HER2*) positive	3/16 (18.8)	9/53 (17)	5/37 (13.5)	4/9 (44.4)	2/7 (28.6)	35/133 (26.3)	50/242 (21.1)	108/497 (21.7)
TNBC	6/17 (35.3)	15/72 (20.8)	11/41 (26.8)	1/22 (4.5)	3/16 (18.8)	31/139 (22.3)	62/229 (27.1)	129/536 (24.1)
Germline variant	70/247 (28.3)	16/89 (18)	4/62 (6.5)	4/61 (6.6)	5/75 (6.7)	10/183 (5.5)	35/298 (11.7)	144/1015 (14.2)
*BRCA1*	63/247 (25.5)	7/89 (7.8)	1/62 (1.6)	0	1/75 (1.3)	1/183 (0.5)	19/298 (6.3)	92/1015 (9.3)
*BRCA2*	6/247 (2.4)	5/89 (5.6)	2/62 (3.2)	2/61 (3.3)	3/75 (4)	3/183 (1.6)	12/298 (4)	33/1015 (3.2)
*PALB2*	ND	4/89 (4.4)	0	2/61 (3.3)	1/75 (1.3)	4/183 (2.2)	2/298 (1)	13/1015 (1.3)
Other^a^	1/247 (0.4)	0	1/62 (1.6)	0	0	2/183 (1.1)	2/298 (0.6)	6/1015 (0.6)

Abbreviations: BC, breast cancer, BMI, body mass index (calculated as weight in kilograms divided by height in meters squared); ER, estrogen receptor; NR, not reported; OC, ovarian cancer; TNBC, triple negative breast cancer.

^a^ Other variants include *ATM*, *CHEK2*, *NBN*, *RAD51C*, *STK11*, and *TP53*.

### Inherited Breast and Ovarian Cancer in Caribbean Populations

A total of 1018 people diagnosed with invasive breast and ovarian cancer were tested for *BRCA1* and *BRCA2*. Subsequent multigene panel testing was conducted in Barbados, Cayman Islands, and Dominica for all patients and in Trinidad, Jamaica, and Haiti for those diagnosed younger than age 40 years. Overall, 9.2% had a P/LP germline variant in *BRCA1*, 3.4% in *BRCA2*, and 1.3% in *PALB2* (Table 1). Only 0.6% had a P/LP variant in moderate risk genes, *RAD51C*, *CHEK2*, *NBN*, *STK11*, and *ATM*. There were no variants found in *MLH1, MSH2, MSH6*, or *PMS2*. The rate of inherited breast and ovarian cancer in the Bahamas was 70 of 247 (28%) with recurrent founder variants *BRCA1* and *BRCA2*. There were 7 founder variants in 92% of variant carriers and a single pathogenic variant was found in the remaining 8% (eTable 2 in the Supplement). In Barbados, 17.4% had P/LP variants in *BRCA1* (1 recurrent variant in 4 unrelated individuals), *BRCA2*, and *PALB2*. In Cayman Islands, 6.5% (4 of 62) had a P/LP variant in *BRCA1, BRCA2*, and *ATM*. In Dominica, 6.6% (4 of 61) of the cohort had a germline variant in *BRCA2* and *PALB2* recurrent variants. In Haiti, 6.7% (5 of 75) of patients with breast cancer had a variant in *BRCA1*, *BRCA*2, or *PALB*2. In Jamaica, we found a 5.5% (10 of 183) rate of inherited breast cancer with germline variants in *BRCA1*, *BRCA2*, *PALB2*, *STK11*, and *NBN* genes. Notably, 9.0% of the deleterious variants were in *PALB2*, making it the highest rate of this variant in the world. In addition, in Trinidad and Tobago, 11.7% had a P/LP variants in the genes tested: *BRCA1* with 2 recurrent variants, *BRCA2* with 3 recurrent variants and *PALB2* with 1 recurrent variant. Other P/LP variants were in *RAD51C* and *CHEK2* each (Figure). In this cohort, 64% of P/LP germline variants were characterized by a frameshift, nonsense, missense, or large deletions in *BRCA1* (10 recurring variants), 23% in *BRCA2* (6 recurring variants), and 9% in *PALB2* (2 recurring variants). Women with invasive breast cancer from Jamaica and Barbados had high rates of *PALB2* P/LP variants at 2.2% and 4.4%, respectively. There were 29 unique variants in *BRCA1* in 92 individuals, 23 unique variants in *BRCA2*, and 11 distinct variants in *PALB2* seen in 13 individuals across 5 countries.

**Figure.**
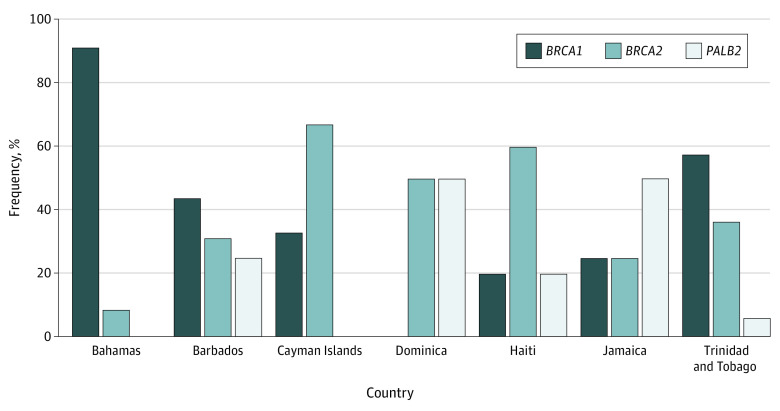
Distribution of Pathogenic and Likely Pathogenic Variants by Country

### Characteristics of Germline Variant Carriers

#### Variants of Unknown Significance

Of the 1018 tested, 47 (4.6%) or 43 of 250 with full panel testing (17.2%) had a VUS (eFigure 1, eTable 2, and eTable 3 in the Supplement). Only individuals identified with breast cancer had a VUS (mean [SD] age, 46.1 [12.5] years). Three women had bilateral breast cancer with VUS in *APC*, *ATM*, and *BRCA2*, 2 of whom were diagnosed before the age of 50 years.

### Pathogenic and Likely Pathogenic Variants

There were 144 variant carriers identified in the cohort. The mean (SD) age of all P/LP variant carriers for breast cancer was 40.7 (9.2) years, significantly younger than those without germline variants (mean [SD] age, 47.5 [10.7] years; *P* = .03). Patients with breast cancer with a *BRCA1* variant had a mean (SD) age of 38.7 [8.5] years, compared with 43.5 [] years for *BRAC2* and 49.9 [5.8] years for *PALB2*. Within the study, the mean BMI (28.2) was not different between variant carriers and noncarriers. Of the P/LP variant carriers, 43.9% (29 of 66) were diagnosed with TNBC compared with 21.1% of the noncarriers (*P* < .001). *BRCA1* positive germline status was associated with higher odds of TNBC (OR, 10.3; 95% CI, 2.05-19.54; *P* = .001). Of the 21 study participants with ovarian cancer, 5 had a P/LP variant in *BRCA1* (n = 4) and *BRCA2* (n = 1) and were diagnosed with high-grade papillary serous ovarian cancer. The mean (SD) age of ovarian cancer variant carriers was 51.4 (10.0) years compared with 46.5 (14.4) years for noncarriers (*P* = .18) (Table 2; eFigure 2 in the Supplement). Of variant carriers, 13.2% (19 of 144, all *BRCA1*) had a second cancer in the contralateral breast or ovarian cancer. One of 4 men had a germline *BRCA2* variant (Table 3).

**Table 2.** Characteristic Differences Between Variant Carriers and Noncarriers

Variable	Variant carriers, No. (%)		*P* value
	Yes (n = 144)	No (n = 871)	
Age, mean (SD)^a^			
BC	40.7 (9.2)	47.5 (10.7)	.03
OC	51.4 (10)^b^	47 (14.2)	.18
BMI, mean (SD)^a^	29.6 (6.4)	29.01 (6.5)	.67
BMI ranges^c^			
<24.9	27/134 (20.1)	220/849 (25.9)	NR
25-29.9	51/134 (38.1)	277/849 (32.6)	NR
>30	56/134 (41.8)	352/849 (41.5)	NR
ER^d^			
Positive	32/67 (47.8)	311/509 (61.1)	.046
Negative	35/67 (52.2)	198/509 (38.9)	NR
*ERBB2* (formerly *HER2*)^d^			
Positive	5/59 (8.5)	103/437 (23.6)	.01
Negative	54/59 (91.5)	334/437 (76.4)	NR
TNBC^d^	29/66 (43.9)	100/469 (21.4)	<.001
Relatives with cancer^d^			
Breast	113/141 (80.1)	395/864 (45.7)	.001
Ovarian cancer	28/137 (20.4)	85/862 (9.9)	<.001
Other types of cancer	7/144 (4.9)	67/871 (7.7)	.85
Prostate	4/28 (14.3)	24/28 (85.7)	NR
Lung	2/12 (16.7)	10/12 (83.3)	NR
Colon	0/9 (11.1)	9/9 (100)	NR
Stomach	1/5 (20)	4/5 (80)	NR
Gynecologic	0	5/5 (100)	NR
Hematologic	0	9/9 (100)	NR
Other types of rare cancer	2/13 (15.4)	11/13 (84.6)	NR

Abbreviations: BC, breast cancer, BMI, body mass index (calculated as weight in kilograms divided by height in meters squared); ER, estrogen receptor; NR, not reported; OC, ovarian cancer; TNBC, triple negative breast cancer.

^a^ Comparison made by independent *t* test.

^b^ Only 5 cases of variant carriers with ovarian cancer.

^c^ BMI cutoffs according to American Cancer Society Guidelines.^21,22^

^d^ Comparisons by χ^2^ test.

**Table 3.** Clinical and Pathological Characteristics Among Pathogenic Variant Carriers

Variable	No. (%)			*P* value
	*BRCA1* (n = 92)	*BRCA2* (n = 33)	*PALB2* (n = 13)	
Age, mean (SD)^a^				
BC	38.7 (8.7)	43.5 (8.5)	49.9 (5.8)	<.001
OC	48.7 (9.3)	62^b^	NR	.30
BMI, mean (SD)^a^	30.4 (6.2)	29.2 (7.6)	27.4 (5.3)	.28
ER^c^				
Positive	6/31 (19.4)	13/21 (61.9)	9/10 (90)	<.001
Negative	25/31 (80.6)	8/21 (38.1)	1/10 (10)	NR
*ERBB2* (formerly *HER2*)^c^				
Positive	0	2/19 (10.5)	1/6 (16.7)	.07
Negative	29/29 (100)	17/19 (89.5)	5/6 (83.3)	NR
TNBC^c^	24/31 (77.4)	5/20 (25)	0	<.001
Relatives with cancer^c^				
BC	72/89 (80.9)	23/33 (69.7)	12/13 (92.3)	.19
OC	22/87 (25.3)	4/33 (12.1)	2/12 (16.7)	.27

Abbreviations: BC, breast cancer, BMI, body mass index (calculated as weight in kilograms divided by height in meters squared); NR, not reported; OC, ovarian cancer; TNBC, triple negative breast cancer.

^a^ Comparison made by 1-way analysis of variance.

^b^ Only 1 *BRCA2* variant carrier with ovarian cancer.

^c^ Comparisons by χ^2^ test.

Half of all participants (50.5%) had a family history of a first-degree or second-degree relative with breast cancer and 11.3% with ovarian cancer. Of individuals with a VUS, 51.1% had a family history of breast cancer and 8.5% of ovarian cancer. Of the 144 probands identified, 80.1% had a family history of breast cancer and 20.4% with ovarian cancer. Any family history of breast cancer was associated with both P/LP *BRCA1* variant (OR, 4.87; 95% CI, 2.82-8.42; *P* < .001) or a P/LP *BRCA2* variant (OR, 3.07; 95% CI, 1.40-6.71; *P* = .005). Increasing numbers of first-degree and second-degree family members with breast cancer were associated with higher odds of having a *BRCA1* (OR, 1.93; 95% CI, 1.68-2.21; *P* < .001) and a *BRCA2* variant (OR, 1.52; 95% CI, 1.26-1.85; *P* < .001). Any family history of ovarian cancer (irrespective of number of affected family members) was associated with a germline *BRCA1* variant (OR, 2.78; 95% CI, 1.59-4.87; *P* < .001) but not *BRCA2* (OR, 1.36; 95% CI, 0.47-3.97; *P* = .57).

## Discussion

This is, to our knowledge, the largest association study evaluating inherited cancer in a cohort of Caribbean patients with breast and ovarian cancer. The study revealed a high rate of inherited variants in patients of young ages with a wide spectrum of variants along the Fanconi anemia pathway (*BRCA1*, *BRCA2*, and *PALB2*). A few of these variants have been previously reported to be of West African, Ashkenazi, or Western European origin, while most have not been previously reported (eTable 2 in the Supplement).

The wide and diverse variety of genomic ancestry found in the Caribbean can be traced to waves of forced immigration in a background of an Indigenous population, including the trans-Atlantic slave trade, European colonization, the importation of both African individuals after the abolition of slavery and indentured laborers from China and the Indian subcontinent. Populations from these areas were moved into islands with substantially different geographies and economies, which may explain, in part, the persistent ancestral patterns of recurrent variants (The Bahamas) and the diversity in others (Trinidad and Tobago).^23^ The African ancestry in the Caribbean is predominantly from West Africa. There, the rates of inherited breast cancer range between 14.1% in Nigeria and 15.2% in Uganda and Cameroon. Rates of inherited breast cancer in African American women range between 12.4% in those 50 years and younger and 22% in those reporting a family history of cancer.^9,10,24,25^ This study’s findings suggest that, irrespective of island of birth, native residents of Caribbean islands have high rates of inherited breast and ovarian cancer.

In Barbados, 18% of patients with breast and ovarian cancer had a germline variant with only 1 recurrent, pathogenic variant in *BRCA1*, which was found in 4 unrelated individuals. In Trinidad, there were 19 variants in *BRCA1* and 12 in *BRCA2*, with only 3 variants shared by 6 individuals. This broad spectrum of variants reflects the diverse population of Trinidad and Tobago, which consists of individuals from South East Asia, East Asia, Africa, and Europe (eFigure 3 in the Supplement). The highest prevalence of inherited breast cancer was found in the Bahamas associated with 7 recurrent variants in the *BRCA1* and *BRCA2*. Only 3 individuals outside of the Bahamas had these recurring variants (those individuals lives in Cayman and Trinidad); therefore the variants identified in the Bahamas are unique to that population and highly penetrant. A 2019 study by Yang et al^26^ reported *PALB2* as a major breast cancer susceptibility gene and found substantial associations between germline pathogenic variants with ovarian cancer and male breast cancer. In our study, women with invasive breast cancer from Jamaica and Barbados had high rates of *PALB2* P/LP variants. This finding further suggests that in the African diaspora rare variants are relatively common and have appreciable effects on cancer risk and population size. The data also highlight that genetic admixture of ancestries influences the rate and diversity in the types of variants identified and indicate use of targeted therapy in countries in the Caribbean with high to middle income.

The VUS rates in this study were approximately 4.6% overall and 17.2% of the 30-gene panel, included women with bilateral breast cancer. Twelve percent to 36% of African Americans and Caribbean nationals have VUS in *BRCA1* and *BRCA2*.^27,28,29,30^ A study from 2018^31^ has reported that expanded multigene panel testing in patients of non-White heritage has increased both the number of genes and overall numbers of VUS classifications.

Although Lynch syndrome is more common than hereditary breast and ovarian cancer syndrome in the general US population, we did not see germline variants in the DNA mismatch repair genes. Very few studies have reported on Lynch syndrome rates in a minority population.^32^ In our study of individuals with primarily African descent, family history of colorectal cancer was rare; therefore, Lynch syndrome is unlikely to be a major factor associated with breast cancer in the Caribbean population.

There is very low uptake of screening mammography in the Caribbean (86% of the breast cancers in this study were self-detected), and the current American Cancer Society^21,22^ and National Comprehensive Cancer Network^33^ guidelines for breast cancer screening are inadequate for this population because a significant proportion develop breast cancer before screening begins. As the cost of panel testing decreases, it may be more feasible to offer panel testing to women with a family history of breast and/or ovarian cancer and then move the women with positive tests into an early and intensive screening cohort, leaving the women without variants in the standard screening model.^34,35^

### Limitations

This study has limitations. Germline genetic testing evolved to panel testing during the course of the study. There was potential selection bias. We advertised across the different countries based on best practices, input from local stakeholders, and clinicians practicing in the individual countries. This approach resulted in successful recruitment of this cohort. In the context of this study, and other populations of predominantly African descent, many women report self-identification of breast lumps, which is a reflection on multiple aspects of health equity such as access to health information and diagnostic/screening services and, therefore, may influence reported rates of inherited cancers. It is a recognized limitation that geographic isolation, influence of genomic ancestry, and evolution of genetic testing approaches from single genes to panel testing all affect reported VUS rates. Due to exhaustion of the Bahamian samples’ cohort, we were unable to apply panel testing to this population. Currently in the Bahamas, patients with newly diagnosed breast and ovarian cancer undergo panel testing for which preliminary data suggest common *BRCA1*, *BRCA2*, and *RAD51C* variants.^36,37^ Because of differences in health systems across the region, participants in certain countries had low reports of ER, PR, and *ERBB2*, which may indicate limited molecular diagnostic testing. We observed discrepancies in the expected mean age of patients in this study compared with local tumor registry data. We postulate that this discrepancy is due to self-selection of the patients who participated in the study with young women being more motivated to participate than older women.

## Conclusions

This genetic association study was a large, unique, and multinational study of breast and ovarian cancer in the Caribbean population. Pathogenic variants in the breast cancer genes of *BRCA1*, *BRCA2*, and *PALB2* are common causes of breast cancer in Caribbean women. Overall, 10% of people of African origin in the US are Caribbean-born,^38^ with 57% coming from Jamaica and Haiti alone,^38^ and this number is predicted to reach 17% by 2060.^39^ People of African descent are understudied and undertested in both the breast and gynecologic cancer settings.^40,41^ Targeted genetic testing of only *BRCA1* and *BRCA2* is insufficient in Caribbean women, and panel (multigene) testing should be recommended.^42^ Adjustment of the threshold recommendations for multigene panel testing in both Caribbean-born individuals and those of Caribbean ancestry might be warranted given the high incidence of pathogenic variants. These data may be useful in screening, increasing awareness of cancer risk, and encouraging risk reduction strategies in people of Caribbean origin and their unaffected family members.^43^ Awareness of the heightened risks among these patients may help minimize morbidity and maximize care in a group already overburdened with well-described cancer health disparities.

## Notes

Supplement. **eTable 1.** Mode of Diagnosis **eTable 2.** Pathogenic Variants Across Cohort in Breast and Ovarian Cancer Patients **eTable 3.** Distribution and Type of Variants of Unknown Significance Across the 7 Countries **eFigure 1.** Oncoprint of Pathogenic and Likely Pathogenic Variants Plotted on the Genes Identified in the Cohort Study **eFigure 2.** A. Frequency of Pathogenic Variant Carriers by Age. B. Frequency of Most Common Pathogenic Variants Genes by Age **eFigure 3.** Distribution of Self-identified Race in Study Population
