# Supplementary material for: Gene Sequencing for Pathogenic Variants Among Adults With Breast and Ovarian Cancer in the Caribbean
Source: JAMA Netw Open. 2021 Mar 1;4(3):e210307. doi: 10.1001/jamanetworkopen.2021.0307 (PMC7921902; doi:10.1001/jamanetworkopen.2021.0307)
Supplement: Supplement. — eTable 1. Mode of Diagnosis eTable 2. Pathogenic Variants Across Cohort in Breast and Ovarian Cancer Patients eTable 3. Distribution and Type of Variants of Unknown Significance Across the 7 Countries eFigure 1. Oncoprint of Pathogenic and Likely Pathogenic Variants Plotted on the Genes Identified in the Cohort Study eFigure 2. A. Frequency of Pathogenic Variant Carriers by Age. B. Frequency of Most Common Pathogenic Variants Genes by Age eFigure 3. Distribution of Self-identified Race in Study Population [file jamanetwopen-e210307-s001.pdf]

Supplemental Online Content

George SHL, Donenberg T, Alexis C, et al. Gene sequencing for pathogenic variants among adults with breast and ovarian cancer in the Caribbean. *JAMA Netw Open*. 2021;4(3):e210307. doi:10.1001/jamanetworkopen.2021.0307

eTable 1. Mode of Diagnosis
eTable 2. Pathogenic Variants Across Cohort in Breast and Ovarian Cancer Patients
eTable 3. Distribution and Type of Variants of Unknown Significance Across the 7 Countries
eFigure 1. Oncoprint of Pathogenic and Likely Pathogenic Variants Plotted on the Genes Identified in the Cohort Study
eFigure 2. A. Frequency of Pathogenic Variant Carriers by Age. B. Frequency of Most Common Pathogenic Variants Genes by Age
eFigure 3. Distribution of Self-identified Race in Study Population

This supplemental material has been provided by the authors to give readers additional information about their work.

© 2021 George SHL et al. *JAMA Network Open*.

eTable 1. Mode of Diagnosis								
Mode of Diagnosis	**Bahamas (247)**	**Barbados (92)**	**Cayman (62)**	**Dominica (61)**	**Haiti (75)**	**Jamaica (183)**	**Trinidad (298)**	**Total (1018)**
**Mammogram** No. (%)	1/16 (6.3)	10/89 (11.2)	11/61 (18)	2/60 (3.3)	0	21/176 (11.9)	20/291 (6.9)	65/765 (8.5)
**Self** No. (%)	12/16 (75)	71/89 (79.8)	44/61 (72.1)	55/60 (91.7)	65/72 (90.3)	148/176 (84.1)	263/291 (90.4)	658/765 (86)
**Other** No (%)	3/16 (18.8)	8/89 (9)	6/61 (9.3)	3/60 (5)	7/72 (4)	7/176 (4)	8/291 (2.7)	42/765 (5.5)

© 2021 George SHL et al. *JAMA Network Open*.

eTable 2. Pathogenic variants across cohort in Breast and Ovarian cancer patients

Gene	No. of Carriers	c.HGVS	Bahamas	Barbados	Cayman Islands	Dominica	Haiti	Jamaica	Trinidad and Tobago	Reported Founder
BRCA1	35	c.4357+1G>A	35							Bahamas, West African
BRCA1	10	c.4611_4612insG	10							
BRCA1	7	c.5324T>G	5		1				1	
BRCA1	5	c.4986+6T>C	4						1	
BRCA1	4	c.?-232_5077+?del, del ex1-17		4						
BRCA1	4	c.815_824dupAGCCATGTGG	4							
BRCA1	2	c.2766delA							2	
BRCA1	2	c.3365_3366delCA		1				1	1	
BRCA1	2	c.4945_4947delAGAinsTTTT							2	
BRCA1	2	c.68_69delAG	2							Ashkenazi Jew
BRCA1	1	c.(5332+1_5333-1)_(5406+1_5407-1)del, del exon 22							1	
BRCA1	1	c.1561G>A	1							
BRCA1	1	c.1636_1654delATGAATATTACTAATAGTG							1	
BRCA1	1	c.1953_1956delGAAA								
BRCA1	1	c.2071delA	1							
BRCA1	1	c.2138C>A							1	
BRCA1	1	c.2389_2390delGA							1	
BRCA1	1	c.2686_2687insA							1	
BRCA1	1	c.3108delT							1	Norway
BRCA1	1	c.3228_3229delAG		1						
BRCA1	1	c.3331_3334delCAAG							1	

© 2021 George SHL et al. *JAMA Network Open*.

BRCA1	1	c.3358_3359delGT	1					1		
BRCA1	1	c.3756_3759delGTCT		1						
BRCA1	1	c.3764_3765insA							1	
BRCA1	1	c.4618_4621delGAAGinsAAA							1	
BRCA1	1	c.4327C>T							1	French Canada
BRCA1	1	c.5177_5180delGAAA							1	African American, Wester European, Asian, Central European
BRCA1	1	c.5510G>A							1	Czech Republic, France
BRCA1	1	c.672delT					1			
**Total**	**92**		**63**	**7**	**1**	**0**	**1**	**2**	**19**	
**BRCA2**	No. of Carriers	c.HGVS	Bahamas	Barbados	Cayman Islands	Dominica	Haiti	Jamaica	Trinidad and Tobago	
BRCA2	4	c.7900delA	4							
BRCA2	3	c.1310_1313delAAGA	1	1						Nigeria - Yoruba
BRCA2	3	c.7558C>T							2	
BRCA2	2	c.5909C>A					1		2	Italian, Western Europe
BRCA2	2	c.632-3C>G		1		1				
BRCA2	2	c.9382C>T							2	

© 2021 George SHL et al. *JAMA Network Open*.

BRCA2	1	c.3865_3868delAAAT								
BRCA2	1	c.1308_1309delGA							1	
BRCA2	1	c.1736T>G		1			1			
BRCA2	1	c.1763_1766delATAA							1	
BRCA2	1	c.1813delA				1				Danish, Japanese, German
BRCA2	1	c.3109C>T							1	
BRCA2	1	c.3599_3600delGT			1					
BRCA2	1	c.476-1G>T						1		
BRCA2	1	c.5073dupA			1					
BRCA2	1	c.6313delA						1		
BRCA2	1	c.6373_6375delACCinsG							1	
BRCA2	1	c.6373delA		1			1			
BRCA2	1	c.6405_6409delCTTAA		1						
BRCA2	1	c.6437_6440delATCA							1	
BRCA2	1	c.7977-1G>A							1	
BRCA2	1	c.7980T>G								
BRCA2	1	c.899_901insGT	1							
**Total**	**33**		**6**	**5**	**2**	**2**	**3**	**2**	**12**	
**PALB2**	No. of Carriers	c.HGVS	Bahamas	Barbados	Cayman Islands	Dominica	Haiti	Jamaica	Trinidad and Tobago	
PALB2	2	c.1571C>G							2	
PALB2	2	c.3350+4A>G				2				
PALB2	1	c.1002C>A		1						
PALB2	1	c.2052delC						1		
PALB2	1	c.2587-1G>C		1						Pakistan

© 2021 George SHL et al. *JAMA Network Open*.

PALB 2	1	c.3166C>T						1		
PALB 2	1	c.3323delA					1			
PALB 2	1	c.43G>T						1		
PALB 2	1	c.751C>T		1						
PALB 2	1	c.758_759insT						1		
PALB 2	1	c.827dup		1						
**Total**	**13**		**0**	**4**	**0**	**2**	**1**	**4**	**2**	
	No. of Carriers	c.HGVS	Bahamas	Barbados	Cayman Islands	Dominica	Haiti	Jamaica	Trinidad and Tobago	
ATM	1	c.6404dup			1					
CHEK 2	1	c.798?_890+?del							1	
NBN	1	c.183delT						1		
RAD51C	1	c.656T>A							1	
STK11	1	c.1371_2611+?del						1		
TP53	1	c.517G>A	1							

© 2021 George SHL et al. *JAMA Network Open*.

eTable 3: Distribution and type of variants of unknown significance across the 7 countries					
Country	Gene Altered	Genetic Testing Result	Specific DNA mutation	Age at 1st Breast Cancer Diagnosis	Age at 2nd Breast Cancer Diagnosis
**Bahamas**	BRCA2	VUS	c.7504C>T	30	
	BRCA2	VUS	c.5640T>G	45	
	BRCA2	VUS	c.6220C>A	44	52
**Barbados**	APC	VUS	c.1610G>A	51	
	ATM	VUS	c.995A>G	40	
	ATM	VUS	c.7174C>T	49	
	ATM	VUS	c.7071G>A	37	45
	ATM	VUS	c.7166C>T	50	
	BARD1	VUS	c.562C>T	53	
	BRCA1	VUS	c.3394A>G	52	
	BRCA2	VUS	c.187A>C	54	
	BRCA2	VUS	c.4624G>A	41	
	BRCA2	VUS	c.9748T>A	51	
	BRCA2	VUS	c.818C>T	45	
	BRIP1	VUS	c.932A>G	70	
	MSH2	VUS	c.814G>A	42	
	NBN	VUS	c.52C>A	39	
	PALB2	VUS	c.2066C>T	52	
	RAD51C	VUS	c.899C>T	51	
**Cayman Islands**					
	APC	VUS	c.5690A>C	61	
	APC	VUS	c.6106G>A	43	
	ATM	VUS	c.1010G>A	25	
	ATM	VUS	c.7174C>T	48	
	ATM	VUS	c.3793T>C	47	
	ATM	VUS	c.7307+18A>G	40	
	BRCA2	VUS	c.7213G>C	47	
	CDH1	VUS	c.1566-15C>A	52	
	CHEK2	VUS	c.1586G>A	50	
	PALB2	VUS	c.3059A>G	70	
	PMS2	VUS	c.1883G>A	52	
	RAD51C	VUS	c.572-14delT	70	

© 2021 George SHL et al. *JAMA Network Open*.

**Dominica**					
	APC	VUS	c.7471A>G	65	
	APC	VUS	c.7540A>G	58	
	ATM	VUS	c.6543G>T	47	
	ATM	VUS	c.25C>T	82	
	BRCA2	VUS	c.3176T>G	51	
	BRCA2	VUS	c.5846A>G	42	
	BRIP1	VUS	c.3196delT	42	
**Haiti**	BRCA1	VUS	c.4181C>T	32	
**Trinidad and Tobago**					
	APC	VUS	c.3445G>A	31	44
	ATM	VUS	c.6418T>C	33	
	BAP1	VUS	c.1058T>C	28	
	BARD1	VUS	c.1314+6A>C	31	
	BARD1	VUS	c.1601C>T	32	
	CHEK2	VUS	c.158C>G	29	
	PALB2	VUS	c.3082G>C	32	
	TP53	VUS	c.749C>T	33	

© 2021 George SHL et al. *JAMA Network Open*.

eFigure 1. Oncoprint of pathogenic and likely pathogenic variants plotted on the genes identified in the cohort study.

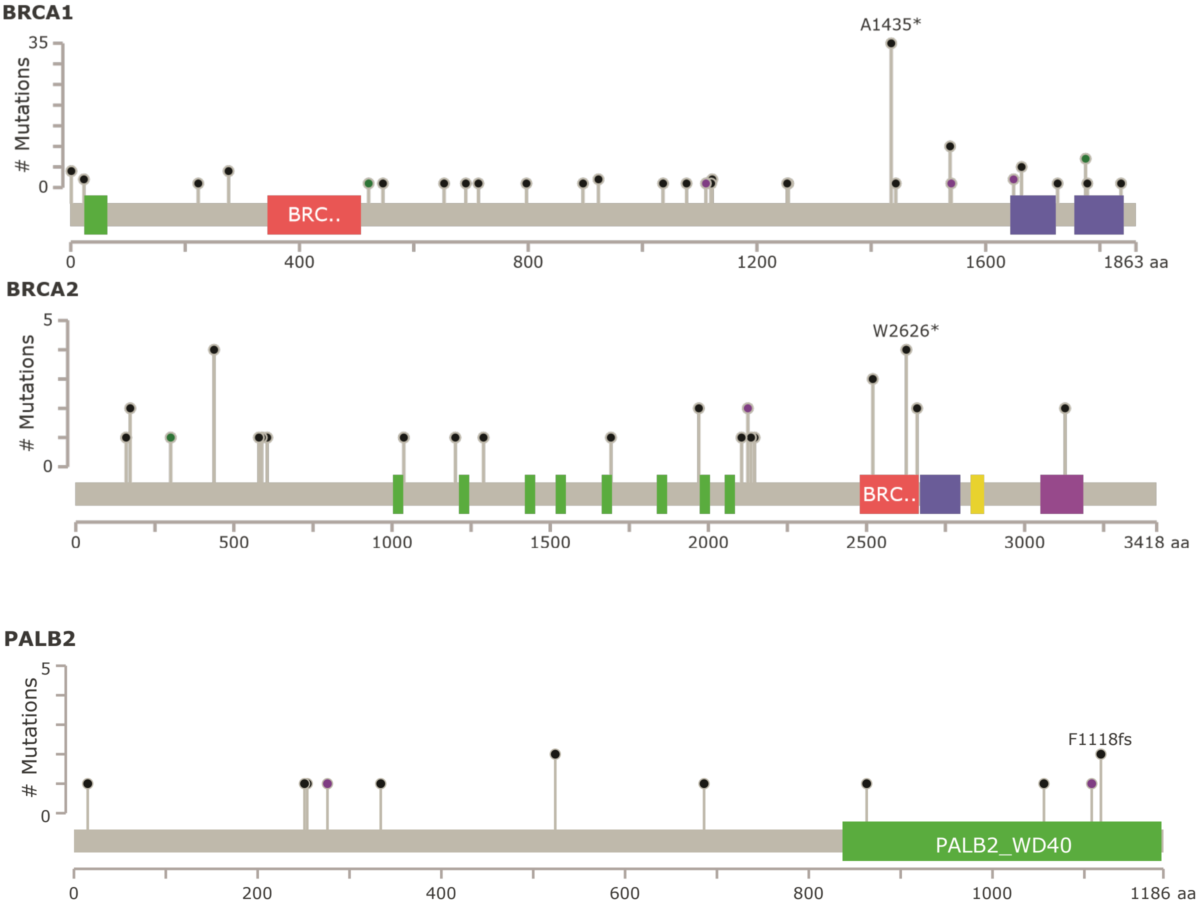

© 2021 George SHL et al. *JAMA Network Open*.

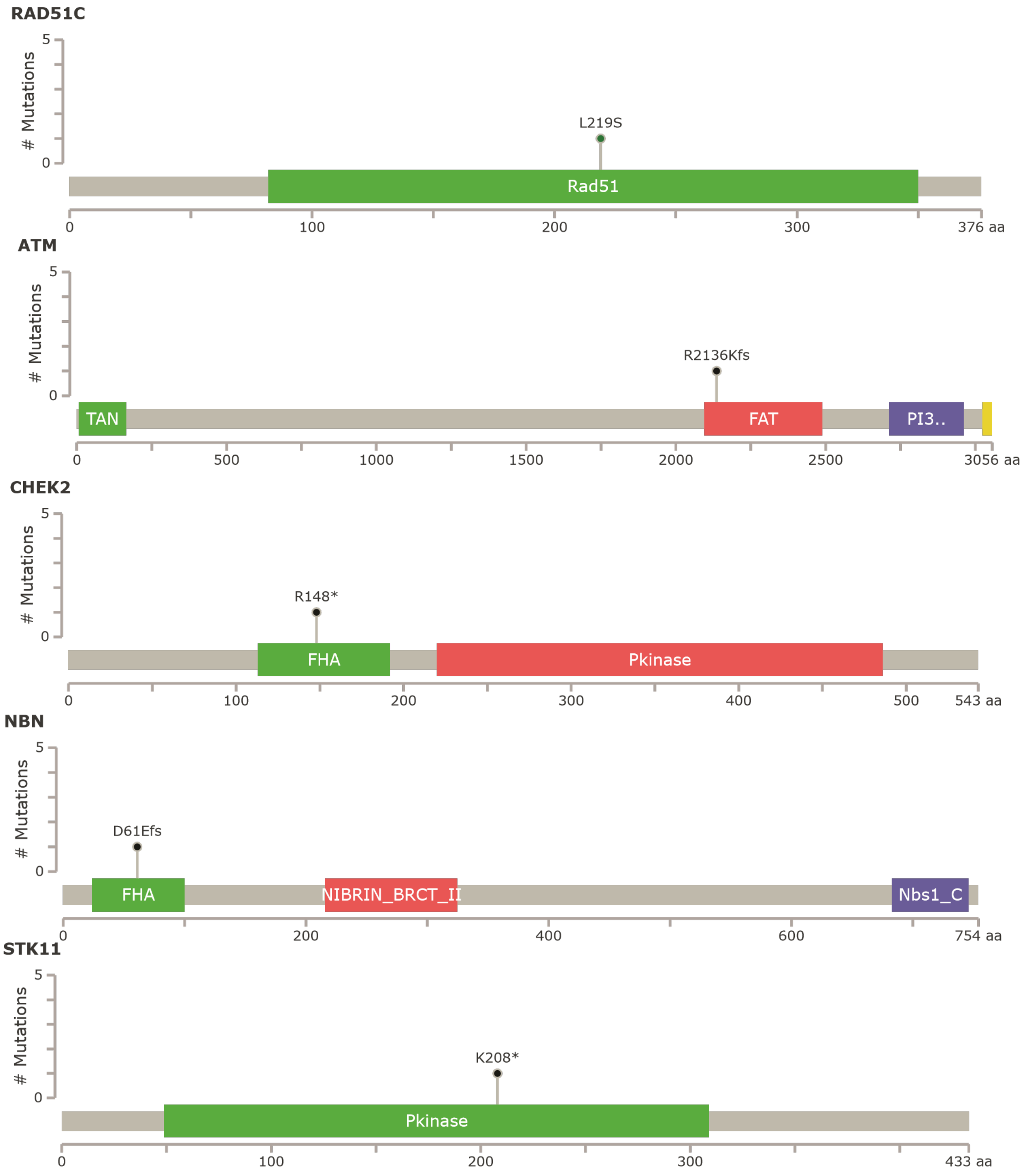

© 2021 George SHL et al. *JAMA Network Open*.

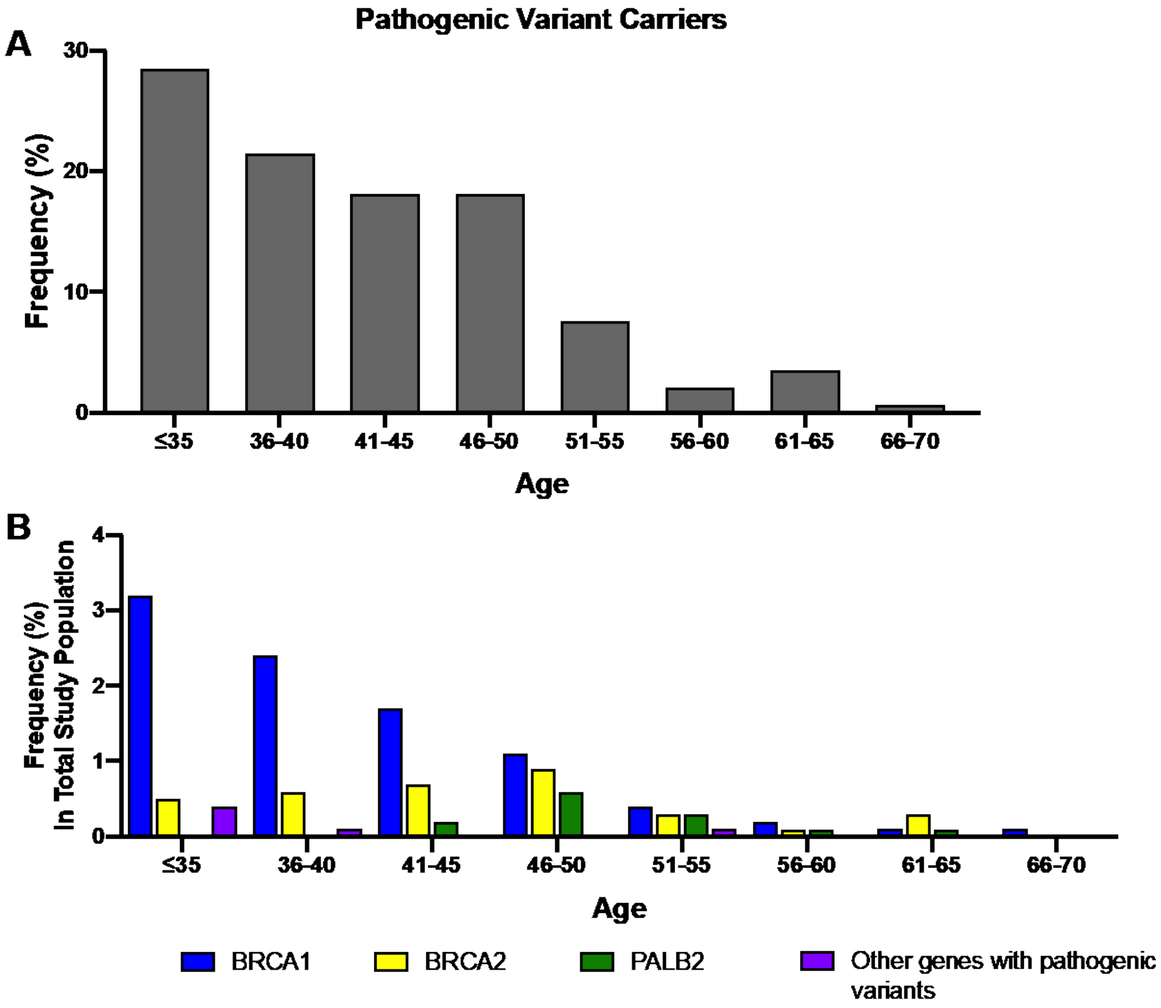

eFigure 2. A. Frequency of pathogenic variant carriers by age. **B.** Frequency of most common pathogenic variants genes by age.

© 2021 George SHL et al. *JAMA Network Open*.

eFigure 3. Distribution of self-identified race in study population.

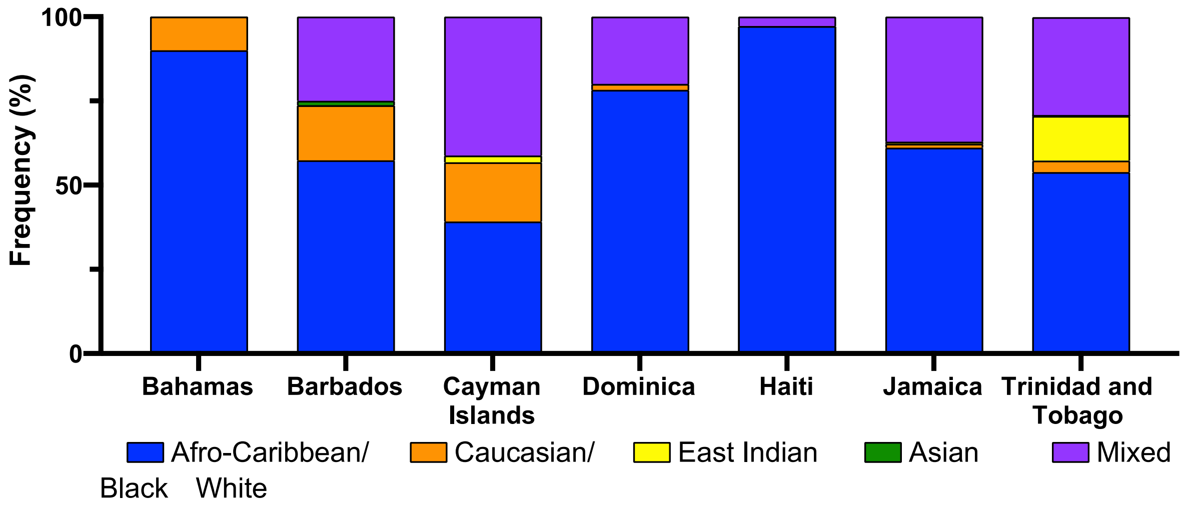

© 2021 George SHL et al. *JAMA Network Open*.
